# Attenuation of prostaglandin E_2 _elimination across the mouse blood-brain barrier in lipopolysaccharide-induced inflammation and additive inhibitory effect of cefmetazole

**DOI:** 10.1186/2045-8118-8-24

**Published:** 2011-10-21

**Authors:** Shin-ichi Akanuma, Yasuo Uchida, Sumio Ohtsuki, Masanori Tachikawa, Tetsuya Terasaki, Ken-ichi Hosoya

**Affiliations:** 1 Department of Pharmaceutics, Graduate School of Medicine and Pharmaceutical Sciences, University of Toyama, 2630 Sugitani, Toyama 939-0364, Japan; 2 Division of Membrane Transport and Drug Targeting, Graduate School of Pharmaceutical Sciences, Tohoku University, Aoba, Aramaki, Aoba-ku, Sendai, Miyagi 980-8578, Japan

**Keywords:** Blood-brain barrier, lipopolysaccharide, inflammation, multidrug resistance-associated protein, MRP4, Oat3, Oatp1a4, PGE_2_, prostaglandin, transporter

## Abstract

**Background:**

Peripheral administration of lipopolysaccharide (LPS) induces inflammation and increases cerebral prostaglandin E_2 _(PGE_2_) concentration. PGE_2 _is eliminated from brain across the blood-brain barrier (BBB) in mice, and this process is inhibited by intracerebral or intravenous pre-administration of anti-inflammatory drugs and antibiotics such as cefmetazole and cefazolin that inhibit multidrug resistance-associated protein 4 (Mrp4/Abcc4)-mediated PGE_2 _transport. The purpose of this study was to examine the effect of LPS-induced inflammation on PGE_2 _elimination from brain, and whether antibiotics further inhibit PGE_2 _elimination in LPS-treated mice.

**Methods:**

[^3^H]PGE_2 _elimination across the BBB of intraperitoneally LPS-treated mice was assessed by the brain efflux index (BEI) method. Transporter protein amounts in brain capillaries were quantified by liquid chromatography-tandem mass spectrometry.

**Results:**

The apparent elimination rate of [^3^H]PGE_2 _from brain was lower by 87%, in LPS-treated mice compared with saline-treated mice. The Mrp4 protein amount was unchanged in brain capillaries of LPS-treated mice compared with saline-treated mice, while the protein amounts of organic anion transporter 3 (Oat3/Slc22a8) and organic anion transporting polypeptide 1a4 (Oatp1a4/Slco1a4) were decreased by 26% and 39%, respectively. Either intracerebral or intravenous pre-administration of cefmetazole further inhibited PGE_2 _elimination in LPS-treated mice. However, intracerebral or intravenous pre-administration of cefazolin had little effect on PGE_2 _elimination in LPS-treated mice, or in LPS-untreated mice given Oat3 and Oatp1a4 inhibitors. These results indicate that peripheral administration of cefmetazole inhibits PGE_2 _elimination across the BBB in LPS-treated mice.

**Conclusion:**

PGE_2 _elimination across the BBB is attenuated in an LPS-induced mouse model of inflammation. Peripheral administration of cefmetazole further inhibits PGE_2 _elimination in LPS-treated mice.

## Background

The blood-brain barrier (BBB), which is formed by the tight junctions of brain capillary endothelial cells, expresses various transporters to regulate exchange of compounds between the brain and the circulating blood. Organic anion transporters such as multidrug resistance-associated protein 4 (Mrp4/Abcc4), organic anion transporter 3 (Oat3/Slc22a8) and organic anion transporting polypeptide 1a4 (Oatp1a4/Slco1a4) are involved in the elimination of endogenous anionic compounds across the BBB [1]. Mrp4 is expressed at the luminal membrane of the BBB [2], while Oat3 is expressed at the abluminal membrane of the BBB [3]. Oatp1a4 is localized in both the abluminal and luminal membranes [4]. Involvement of Oat3 and Oatp1a4 in the trans-BBB elimination of various endogenous anionic compounds, such as homovanillic acid and 24S-hydroxycholesterol, has been demonstrated [3,5]. Moreover, we reported that prostaglandin E_2 _(PGE_2_), which is an inflammatory mediator, is eliminated across the BBB in normal mice, and this elimination process involves Mrp4 [6]. Because PGE_2_-metabolizing activity was not detected either in rat brain or cerebral microvessels [7], PGE_2 _elimination across the BBB is considered to be critical for controlling PGE_2 _function in the brain.

Excess PGE_2 _promotes excitatory signal transduction in the brain, leading to fever progression and behavioral abnormalities [8,9]. The concentration of PGE_2 _in the brain is increased by endotoxin challenge during infection [10,11]. Lipopolysaccharide (LPS), a gram-negative bacterial cell surface proteoglycan, is one such endotoxin [11]. Intraperitoneal administration of LPS at the dose of more than 1 mg/kg induces systemic inflammatory response syndrome, including systemic and cerebral increase of inflammatory cytokines and the changes of body temperature and locomotive activity [12,13]. Moreover, LPS at doses of more than 1 mg/kg increases the PGE_2 _concentration in the brain to 28-141 nM, which is 100- to 700-fold greater than normal [14,15]. Although systemic or intracerebral administration of LPS induces PGE_2_-producing enzymes, such as cyclooxygenase (COX)-2 and microsomal prostaglandin E synthase (PGES), in the brain [16-18], attenuation of PGE_2 _elimination across the BBB may also contribute to the dramatic increase of brain PGE_2 _level induced by LPS.

In patients with gram-negative bacterial infection, anti-inflammatory drugs and antibiotics are administered for palliative care and treatment [19,20]. We have reported that the elimination of PGE_2 _across the BBB was inhibited by either intracerebral or intravenous administration of antibiotics, such as cefmetazole and cefazolin, and also by intracerebral administration of non-steroidal anti-inflammatory drug (NSAIDs), such as indomethacin and ketoprofen. These drugs strongly inhibit human MRP4-mediated PGE_2 _transport activity, and Mrp4 is involved in elimination of PGE_2 _across the BBB [6]. Oat3 accepts PGE_2 _as a substrate [21]. Oatp1a4 and prostaglandin transporter (Pgt/Slco2a1) were reported to be involved in prostaglandin E_1 _transport from the circulating blood to the brain [22]. These results raise the possibility that administration of antibiotics and/or NSAIDs could facilitate the increase of PGE_2 _levels in the brain by interaction at organic anion transport system(s) of the BBB, thereby increasing the probability of cerebral adverse effects. Indeed, fever is related to increased PGE_2 _in the brain [8], and is a known adverse effect of cefmetazole [23]. Thus, it is important to examine the effect of these drugs on PGE_2 _elimination across the BBB in inflammation, in order to establish appropriate drug treatment of infectious diseases.

Therefore, the purpose of this study was to investigate the effect of LPS-induced inflammation on PGE_2 _elimination from the brain across the BBB, and to examine whether antibiotics further inhibit the PGE_2 _elimination in LPS-treated mice, using the brain efflux index (BEI) method.

## Methods

### Reagents

Prostaglandin E_2_, [5,6,8,11,12,14,15-^3^H(N)]- ([^3^H]PGE_2_; 185.6 Ci/mmol) was purchased from PerkinElmer Life and Analytical Sciences (Boston, MA, USA). Inulin carboxyl, [carboxyl-^14^C]- ([^14^C]inulin; 1.9 mCi/g) was purchased from Moravek Biochemicals (Brea, CA, USA). Cefazolin sodium salt, cefmetazole sodium salt, cefotaxime sodium salt, cefsulodin sodium salt hydrate, ceftriaxone disodium salt, indomethacin, ketoprofen and LPS (from *E. coli *serotype O111:B4) were obtained from Sigma-Aldrich (St. Louis, MO, USA). Cefotiam was purchased from The United States Pharmacopeial Convention (Kansas City, MO, USA). All other chemicals were commercial reagent-grade products.

### Animals

Adult male C57BL/6J mice (20-30 g) and ddY mice (25-40 g) were purchased from Japan SLC (Hamamatsu, Japan). All experiments conformed to the provisions of the Animal Care Committee, University of Toyama, and were approved by the Animal Care Committee, Graduate School of Pharmaceutical Sciences, Tohoku University. In order to obtain the mouse model of inflammation (LPS-treated mice), 3.0 mg/kg LPS was intraperitoneally administered to mice 24 hours prior to experiments.

### Mouse brain efflux index (BEI) method

*In vivo *mouse brain elimination experiments were performed using the intracerebral microinjection technique [6]. In brief, a C57BL/6J mouse was anesthetized by intraperitoneal administration of pentobarbital (50 mg/kg body weight) and placed in a stereotaxic frame (SR-5M; Narishige, Tokyo, Japan). The applied solution, consisting of [^3^H]PGE_2 _(96 nCi) and [^14^C]inulin (4.8 nCi) dissolved in 0.3 μL extracellular fluid (ECF) buffer (122 mM NaCl, 25 mM NaHCO_3_, 3 mM KCl, 1.4 mM CaCl_2_, 1.2 mM MgSO_4_, 0.4 mM K_2_HPO_4_, 10 mM D-glucose and 10 mM HEPES, pH 7.4) with 0.1% ethanol in the presence or absence of unlabeled compounds, was administered into the secondary somatosensory cortex (S2) region of the brain. The needle was left in the injection configuration for an additional 4 min to prevent reflux of the injected solution along the injection track, before being slowly retracted. [^14^C]Inulin is an impermeable marker used to normalize the actual injection volume, as the injection volume is small (0.3 μL). In the pre-administration study, inhibitor solution (10 μL) at the indicated concentration in ECF buffer with or without 0.25% dimethyl sulfoxide (DMSO) was injected into the S2 region 5 min prior to administration of the applied solution. To examine the effects of benzylpenicillin and digoxin, inhibitor solution containing 100 μM benzylpenicillin or 20 μM digoxin was injected into the S2 region. As a control, ECF buffer with or without 0.25% DMSO was injected. For the intravenous administration study, 200 μL of Ringer-HEPES buffer (141 mM NaCl, 4 mM KCl, 2.8 mM CaCl_2 _and 10 mM HEPES-NaOH, pH 7.4) containing cefmetazole or cefazolin was injected into the jugular vein 15 min prior to administration of the applied solution. To test the effect of amiodarone administration, 20 mg/kg amiodarone was intravenously administered 5 min prior to intravenous administration of cefmetazole or cefazolin. At a designated time, the mouse was decapitated and the left and right cerebrum and cerebellum were excised. Each tissue was dissolved in 2 N NaOH (2 mL) at 55°C for 3 h, and then mixed with 14 mL Hionic-Fluor (PerkinElmer Life & Analytical Sciences). The radioactivity was measured in a liquid scintillation counter equipped with an appropriate crossover correction for^3^H and^14^C (LSC-5100, Aloka, Tokyo, Japan).

The BEI value was defined according to Eqn. 1 and the percentage of [^3^H]PGE_2 _remaining in the ipsilateral cerebrum (100-BEI) was determined using Eqn. 2:

(1) $$\text{BEI}\;\left(\%\right)=\frac{\text{Test}\;\text{substrate}\;\text{undergoing}\;\text{efflux}\;\text{at}\;\text{the}\;\text{BBB}}{\text{Test}\;\text{substrate}\;\text{injected}\;\text{into}\;\text{the}\;\text{brain}}\times 100$$

(2) $$100-\text{BEI}\;\;\left(\%\right)=\frac{\left(\text{Amount}\;\text{of}\;{[}^{3}\text{H}]\text{PG}{\text{E}}_{2}\;\text{in}\;\text{the}\;\text{brain}∕\text{amount}\;\text{of}\;{[}^{14}\text{C}]\;\text{inulin}\;\text{in}\;\text{the}\;\text{brain}\right)}{\left(\text{Concentration}\;\text{of}\;{[}^{3}\text{H}]\text{PG}{\text{E}}_{2}\;\text{in}\;\text{injectate}∕\text{concentration}\;\text{of}\;{[}^{14}\text{C}]\text{inulin}\;\text{in}\;\text{injectate}\right)}\times 100$$

As can be seen from these equations, increase of the 100-BEI value of [^3^H]PGE_2 _indicates a decrease of PGE_2 _elimination rate from the brain across the BBB.

The apparent elimination rate constant (*k_e_*) was determined by fitting a semi-logarithmic plot of 100-BEI, i.e., the percentage remaining in the ipsilateral cerebrum, versus time, using a nonlinear least-squares regression analysis program (MULTI) [24]. To evaluate the inhibitory effect on [^3^H]PGE_2 _efflux across the BBB, the BEI value of [^3^H]PGE_2 _at 40 min was determined in the presence or absence of various drugs.

### Preparation of brain capillary fraction from saline-treated and 3.0 mg/kg LPS-treated mice

Mouse brain capillary fraction was prepared as described [25]. All procedures were carried out at 4°C except for perfusion. Mice were transcardially perfused with phosphate-buffered saline to remove blood under anesthesia induced with pentobarbital, and then the cerebrum was isolated. 20 cerebrums were dissected into 1-mm pieces, and homogenized with a Potter-Elvehjem homogenizer using 10 up and down no-rotated strokes by hand in 4-fold volume solution B (101 mM NaCl, 4.6 mM KCl, 2.5 mM CaCl_2_, 1.2 mM KH_2_PO_4_, 1.2 mM MgSO_4_, 15 mM HEPES, pH 7.4) of brain weight. After the dextran (Mw 60,000-90,000, Sigma-Aldrich) was added to the homogenate as the final concentration of 16%, the mixture was centrifuged at 4500 × g for 10 min. The pellet was suspended in solution A (solution B containing 25 mM NaHCO_3_, 10 mM glucose, 1 mM pyruvate and 5 g/L bovine serum albumin) and passed through a 85 μm nylon mesh, and the filtrate was passed over a column containing 350-500 μm glass beads, and then washed with 50 mL solution A. The brain capillaries adhering to the beads were detached to solution A by gentle agitation, and, as soon as the beads sank, the supernatant including the capillaries was collected. The supernatant was centrifuged at 230 × g for 5 min. The pellet was suspended with solution B, and centrifuged at 1700 × g for 5 min. Again, the pellet was suspended with solution B, and centrifuged at 1700 × g for 5 min. The pellet was suspended with hypotonic buffer (10 mM Tris-HCl, 10 mM NaCl, 1.5 mM MgCl_2_, pH 7.4) and sonicated. The capillaries were stored at -80°C after measurement of the protein concentration by the Lowry method using the DC protein assay reagent (Bio-Rad, Hercules, CA, USA).

### Quantification of transporter protein in mouse brain capillaries by LC-MS/MS

The absolute amount of transporter protein in mouse brain capillary endothelial cells was determined by mass spectrometric analysis [26]. The brain capillary fraction (50 μg protein) was suspended in 500 mM Tris-HCl (pH 8.5), 7 M guanidium hydrochloride and 10 mM EDTA, and the proteins were S-carbomoylmethylated. The alkylated proteins were precipitated with a mixture of methanol and chloroform. The precipitates were dissolved in 6 M urea in 100 mM Tris-HCl (pH 8.5), diluted 5-fold with 100 mM Tris-HCl (pH 8.5) and treated with TPCK-treated trypsin (Promega, Madison, WI, USA) at an enzyme/substrate ratio of 1:100 at 37°C for 16 h. The tryptic digests were mixed with isotope-labeled peptides as internal standards (Table 1) and formic acid, and then centrifuged at 4°C and 17,360 × g for 5 min. The supernatants were subjected to LC-MS/MS analysis.

**Table 1:** Peptide probe sequences, selected ions for quantification and limit of quantification in isolated brain capillaries of each protein with LC-MS/MS.

				MRM transition					
					
Accession No.	Synonym (gene name)	Probe sequence	Peptide mass	Q1	Q3-1	Q3-2	Q3-3	Q3-4	**Limit of quantification(fmol/μg)**
Q3TZN9	Mrp4	APVLFFDR	963.5	482.8	796.4	697.4	584.3	437.2	0.0460
	(*Abcc4*)	APVL*FFDR	970.5	486.3	803.4	704.4	584.3	437.2	

O88909	Oat3	YGLSDLFR	969.5	485.8	807.4	750.4	637.3	550.3	0.163
	(*Slc22a8*)	YGLSDL*FR	976.5	489.3	814.4	757.4	644.3	557.3	

Q9EP96	Oatp1a4	EVATHGVR	867.5	434.7	640.4	569.3	468.3	331.2	0.441
	(*Slco1a4*)	EVATHGV*R	873.5	437.7	646.4	575.5	474.3	337.2	

Q9EPT5	Pgt	IFVDYGR	869.0	435.2	756.4	609.3	510.2	395.2	0.312
	(*Slco2a1*)	IFV*DYGR	875.0	438.2	762.4	615.3	510.2	395.2	

The selected peptides were synthesized and their purity was checked with HPLC-UV according to the previous report [26]. The MRM transitions were determined from MS/MS spectra obtained by direct infusion of 1 μM peptide solution at a flow rate of 5 μL/min with a syringe pump (Harvard) into the mass spectrometer. Doubly charged precursor ions were selected (Q1). Four transitions per peptide (Q3-1, -2, -3 and -4), corresponding to high-intensity fragment ions, were selected. The declustering potentials and collision energies were optimized to maximize signal strength. For the stable isotope-labeled peptides, precursor ions and transitions corresponding to those of the unlabeled peptides were selected, with the same declustering potentials and collision energies as for the unlabeled peptides. Bold letters with asterisks indicate amino acid residues labeled with stable isotope (^13^C and^15^N). The limit of quantification was defined as the protein expression level which would give a peak area count of 5000 in the chromatogram when a brain capillary sample is measured by LC-MS/MS.

The LC-MS/MS analysis was performed by coupling an Agilent 1100 HPLC system (Agilent Technologies, Santa Clara, CA, USA) to a triple quadrupole mass spectrometer (API5000; Applied Biosystems, Foster City, CA, USA) equipped with Turbo V ion source (Applied Biosystems). Samples equivalent to 33.3 μg protein were injected onto a Waters XBridge BEH130 C18 (1.0 × 100 mm, 3.5 μm) column together with 500 fmol of isotope-labeled peptides. Mobile phases A and B consisted of 0.1% formic acid in water and 0.1% formic acid in acetonitrile, respectively. The peptides were separated and eluted from the column at room temperature using a linear gradient with a 120-min run time at a flow rate of 50 μL/min. The sequence was as follows: (A:B), 99:1 for 5 min after injection, 50:50 at 55 min, 0:100 at 56 min and up to 58 min, 99:1 at 60 min and up to 120 min.

The eluted peptides were simultaneously and selectively detected by means of electro-spray ionization in a multiplexed multiple reaction monitoring (MRM) mode, which can quantify many molecules simultaneously. The dwell time was 10 msec per MRM transition. Each molecule was monitored with four sets of MRM transitions (Q1/Q3-1, Q1/Q3-2, Q1/Q3-3, Q1/Q3-4) derived from one set of unlabeled and isotope-labeled peptides (Table 1). Chromatogram ion counts were determined by using the data acquisition procedures in Analyst software version 1.4.2 (Applied Biosystems). Signal peaks with a peak area count of over 5000 detected at the same retention time as an isotope-labeled peptide were defined as positive. When positive peaks were observed in three or four sets of MRM transitions, the molecules were considered to be expressed in brain capillaries, and then the protein expression amounts were determined as the average of three or four quantitative values. The limit of quantification (fmol/μg protein) of non-detected molecules in the isolated mouse brain capillary was defined as described previously [25] (Table 1). In brief, the limit of quantification was defined as the protein expression level which would give a peak area count of 5000 in the chromatogram when a brain capillary sample is measured by LC-MS/MS.

### Data analysis

The apparent elimination rate constant (*k_e_*) is presented as the mean ± S.D.. Other data are given as mean ± S.E.M.. Statistical significance of differences between means was determined by means of the unpaired two-tailed Student's *t*-test for two groups and one-way analysis of variance followed by Dunnett's test for more than two groups.

## Results

### Effect of intraperitoneal pre-administration of LPS on PGE_2 _elimination from mouse brain

**Figure 1:**
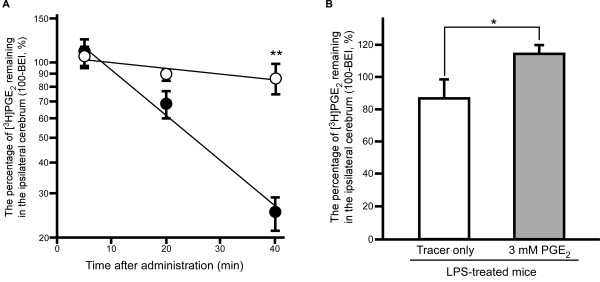
**Effect of LPS treatment on elimination of [^3^H]PGE_2 _from mouse brain**. (A) Time-course of [^3^H]PGE_2 _in the ipsilateral cerebrum after intracerebral microinjection in LPS-treated mice (open circles) and saline-treated mice (closed circles). A mixture of [^3^H]PGE_2 _(96 nCi) and [^14^C]inulin (4.8 nCi; internal reference) dissolved in 0.3 μL ECF buffer containing 0.1% ethanol was injected into the S2 region of mouse brain. Each point represents the mean ± SEM (n = 4 - 5). **p < 0.01, significantly different from the saline-treated mice. (B) Effect of unlabeled PGE_2 _on the percentage of [^3^H]PGE_2 _remaining in the ipsilateral cerebrum at 40 min after intracerebral microinjection in LPS-treated mice. A mixture of [^3^H]PGE_2 _and [^14^C]inulin dissolved in 0.3 μL ECF buffer containing 3 mM PGE_2 _was injected. Each column represents the mean ± S.E.M. (n = 3 - 4). *p < 0.05, significantly different from the tracer only.

Figure 1A shows the time-profile of the percentage of [^3^H]PGE_2 _remaining in the ipsilateral cerebrum after microinjection into the S2 region of LPS-treated mouse brain. The percentage of [^3^H]PGE_2 _remaining decreased in a time-dependent manner in both saline-treated mice and LPS-treated mice. The remaining percentage at 40 min was 3.46-fold greater in LPS-treated mice than in saline-treated mice (*p *< 0.01), indicating that the elimination rate was decreased in the brain of LPS-treated mice. The apparent elimination rate constant (*k_e_*) of [^3^H]PGE_2 _calculated from the slope was 5.52 × 10^-3 ^± 2.67 × 10^-3 ^min^-1 ^in LPS-treated mice (mean ± S.D.), which was significantly lower, by 87.0%, than that in saline-treated mice (4.25 × 10^-2 ^± 0.48 × 10^-2 ^min^-1^, mean ± S.D.) (*p *< 0.01). No radioactivity associated with this elimination process was detected in the contralateral cerebrum or cerebellum (data not shown).

The percentage recovery from brain of [^14^C]inulin, a non-permeable marker, was 50.9 ± 11.3% (n = 5), 31.0 ± 7.5% (n = 3) and 38.8 ± 6.4% (n = 3) at 5 min, 20 min and 40 min, respectively, after microinjection in LPS-treated mice. There was no significant difference between these values. Moreover, the corresponding values in saline-treated mice were 45.5 ± 14.2% (n = 3), 32.8 ± 5.7% (n = 3) and 56.7 ± 10.4% (n = 6) at 5 min, 20 min and 40 min after microinjection, respectively. There was no significant difference in percentage recovery at each time between LPS-treated mice and saline-treated mice.

In LPS-treated mice, intracerebral co-administration of 3 mM unlabeled PGE_2 _significantly increased the percentage of [^3^H]PGE_2 _remaining in the brain at 40 min after microinjection (114 ± 5%) compared to that in the absence of unlabeled PGE_2 _(*p *< 0.05, Figure 1B). This value was nearly identical to the y-intercept of the time-profile of the percentage of [^3^H]PGE_2 _remaining in LPS-treated mice (108 ± 10% (mean ± S.D.), Figure 1A).

### Effect of intraperitoneal pre-administration of LPS on protein expression of organic anion transporters in mouse brain capillaries

The transporter protein expression levels of Mrp4, Oat3, Oatp1a4 and Pgt in mouse brain capillaries were simultaneously quantified by LC-MS/MS (Table 2). In the brain capillaries of LPS-treated mice, Mrp4 protein expression was not significantly altered. However, the protein expression levels of Oat3 and Oatp1a4 in the brain capillaries were significantly decreased by 26% (*p *< 0.05) and 39% (*p *< 0.01), respectively, in LPS-treated mice compared to saline-treated mice. It is reported that Pgt is expressed in rat cerebral endothelial cells [35]. However, we found the protein expression level of Pgt in the brain capillaries was below the detection limit in both LPS-treated and saline-treated mice, indicating that protein expression level of Pgt in mouse brain capillaries is lower than that of Mrp4, Oat3 and Oatp1a4.

**Table 2:** Expression level of organic anion transporters in brain capillaries of LPS-treated mice and saline-treated mice.

	Protein expression level (fmol/μg protein)		
		
Transporter	Saline	3 mg/kg LPS	Ratio (LPS/Saline)
Mrp4	1.80 ± 0.20	1.50 ± 0.13	0.833
Oat3	1.25 ± 0.08	0.929 ± 0.068*	0.743
Oatp1a4	2.46 ± 0.31	1.50 ± 0.14**	0.610
Pgt	N.D.	N.D.	-

Brain capillary-enriched fraction was prepared from ddY mice intraperitoneally injected with 3 mg/kg LPS or saline. The fraction was digested with trypsin and the digest was injected into LC-MS/MS together with internal standard peptides. Each value represents the mean ± S.E.M. (n = 4). *p < 0.05 and **p < 0.01, significantly different from the saline-treated mice. N.D: not detected.

### Effect of intracerebral pre-administration of NSAIDs or cephalosporins on PGE_2 _elimination from brain of LPS-treated mice

The effects of intracerebral pre-administration of NSAIDs or cephalosporins on [^3^H]PGE_2 _elimination from the brain of LPS-treated mice were investigated. As shown in Table 3, the percentage of [^3^H]PGE_2 _remaining in the ipsilateral cerebrum in LPS-treated mice was significantly increased by 1.46- and 1.49-fold by intracerebral pre-administration of cefmetazole (5 mM) and cefotaxime (5 mM), respectively, compared with the LPS-treated control (*p *< 0.05). In contrast, ceftriaxone, cefazolin, indomethacin or ketoprofen had no significant effect on the level of [^3^H]PGE_2 _remaining in the ipsilateral cerebrum of LPS-treated mice, although these drugs were reported to show an inhibitory effect on [^3^H]PGE_2 _elimination in mice not treated with LPS [6]. Intracerebral pre-administration of cefsulodin and clarithromycin, which did not inhibit PGE_2 _elimination from the brain of mice not treated with LPS [6], had no significant effect on the level of [^3^H]PGE_2 _remaining in the brain of LPS-treated mice.

**Table 3:** Effect of intracerebral pre-administration of antibiotics and NSAIDs (5 mM) on [^3^H]PGE_2 _elimination from brain across the BBB in LPS-treated mice.

Compound	Percentage of [^3^H]PGE_2 _remaining in ipsilateral cerebrum (100-BEI, %)	Percentage of control
***Cephalosporins***		
Control	91.9 ± 6.6	100
Cefmetazole	133 ± 16*	146
Cefotaxime	136 ± 1*	149
Ceftriaxone	113 ± 11	124
Cefsulodin	94.2 ± 15.3	103
Cefazolin	85.7 ± 6.2	94.0

***Other antibiotics and NSAIDs***		
Control (0.25% DMSO)	97.2 ± 11.1	100
Indomethacin (0.25% DMSO)	114 ± 9	117
Ketoprofen (0.25% DMSO)	82.8 ± 5.8	85.2
Clarithromycin (0.25% DMSO)	82.2 ± 16.4	84.6

Each compound or ECF buffer with or without 0.25% DMSO (control) was administered at 5 min prior to administration of [^3^H]PGE_2 _in the brain of LPS-treated mice. The percentage of [^3^H]PGE_2 _in the ipsilateral cerebrum was determined at 40 min after intracerebral [^3^H]PGE_2 _microinjection. Each value represents the mean ± S.E.M. (n = 3-8). *p < 0.05, significantly different from control.

### Inhibitory effect of intravenous pre-administration of cefmetazole or cefazolin on PGE_2 _elimination from brain of LPS-treated mice

**Figure 2:**
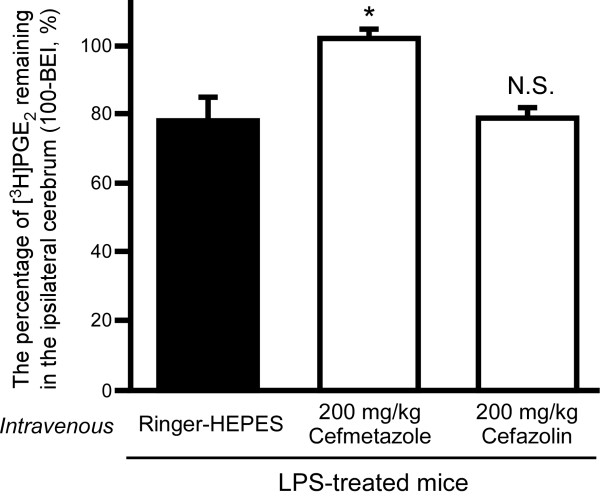
**Effect of intravenous pre-administration of cefmetazole or cefazolin on [^3^H]PGE_2 _elimination from the brain in LPS-treated mice**. Cefmetazole (200 mg/kg), cefazolin (200 mg/kg) or Ringer-HEPES (control) was administered intravenously 15 min prior to administration of [^3^H]PGE_2 _in LPS-treated mice. The 100-BEI value was determined 40 min after intracerebral microinjection of [^3^H]PGE_2_. Each column represents the mean ± S.E.M. (n = 3). *p < 0.05, significantly different from control.

The effects of intravenous pre-administration of cefmetazole or cefazolin on [^3^H]PGE_2 _elimination from the brain of LPS-treated mice were investigated. As shown in Figure 2, the intravenous pre-administration of 200 mg/kg cefmetazole significantly increased the percentage of [^3^H]PGE_2 _remaining (102 ± 3%) in LPS-treated mice (*p *< 0.05), compared with saline pre-administered LPS-treated mice (78.1 ± 6.7%). In contrast, intravenous administration of 200 mg/kg cefazolin had no significant effect (78.4 ± 2.4%).

### Involvement of Oat3 and/or Oatp1a4 in the inhibitory effect of cefmetazole and cefazolin on PGE_2 _elimination from mouse brain

**Figure 3:**
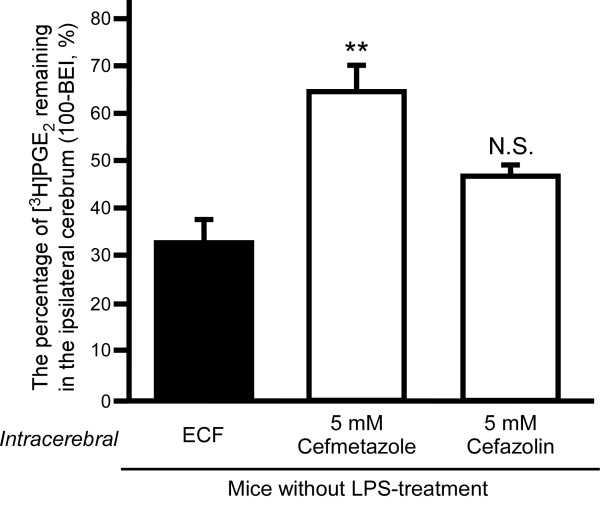
**Inhibitory effect of intracerebral pre-administration of cefmetazole or cefazolin on [^3^H]PGE_2 _elimination from the brain of LPS-untreated mice in the presence of benzylpenicillin and digoxin**. Either cefmetazole (5 mM) or cefazolin (5 mM) or ECF buffer (ECF, control) was co-administered with 100 μM benzylpenicillin and 20 μM digoxin to the S2 region 5 min prior to administration of [^3^H]PGE_2 _in the brain of mice without LPS treatment. The 100-BEI value was determined 40 min after intracerebral microinjection of [^3^H]PGE_2_. Each column represents the mean ± S.E.M. (n = 3). **p < 0.01, significantly different from control.

To investigate the possible involvement of Oat3 and Oatp1a4 in the inhibitory effect of intracerebral pre-administration of cefmetazole and cefazolin on PGE_2 _elimination from the brain, Oat3 and Oatp1a4 were selectively inhibited with benzylpenicillin (100 μM) and digoxin (20 μM), respectively, in the brain of mice not treated with LPS (Figure 3). In the case of intracerebral pre-administration of ECF buffer with benzylpenicillin and digoxin (control), the percentage of [^3^H]PGE_2 _remaining was 32.8 ± 4.8%, which is similar to the value reported for pre-administration of ECF buffer alone (34.7%) [6]. When cefmetazole was pre-administered with benzylpenicillin and digoxin in the brain, the percentage of [^3^H]PGE_2 _remaining was increased by 1.95-fold compared to the control (*p *< 0.01). Intracerebral pre-administration of cefazolin with benzylpenicillin and digoxin did not significantly affect the percentage of [^3^H]PGE_2 _remaining. These results suggest involvement of Oat3 and/or Oatp1a4 in the inhibitory effect of cefazolin in the brain.

### Involvement of Oatp1a4 in the inhibitory effect of cefmetazole and cefazolin in the circulation on PGE_2 _elimination from mouse brain

**Figure 4:**
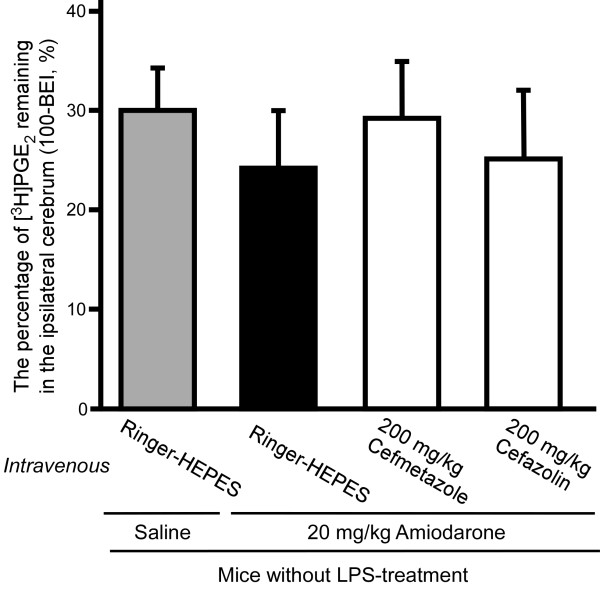
**Inhibitory effect of intravenous pre-administration of cefmetazole or cefazolin on [^3^H]PGE_2 _elimination from the brain of LPS-untreated mice in the presence of amiodarone**. Either cefmetazole (200 mg/kg), cefazolin (200 mg/kg) or Ringer-HEPES were administered with amiodarone (20 mg/kg) or saline intravenously prior to intracerebral administration of [^3^H]PGE_2 _in mice not treated with LPS. The 100-BEI value was determined 40 min after intracerebral microinjection of [^3^H]PGE_2_. Each point represents the mean ± S.E.M. (n = 3 - 4).

To investigate the involvement of Oatp1a4 in the effect of intravenous administration of cefmetazole and cefazolin on PGE_2 _elimination from the brain, Oatp1a4 was inhibited by intravenous administration of amiodarone in mice not treated with LPS. Intravenous administration of amiodarone (20 mg/kg) did not affect the percentage of [^3^H]PGE_2 _remaining in the ipsilateral cerebrum (24.6 ± 5.5%) compared with saline-only treatment (29.9 ± 4.3%). In the case of 20 mg/kg amiodarone intravenous administration, intravenous administration of 200 mg/kg cefmetazole or 200 mg/kg cefazolin did not cause a significant increase in the percentage of [^3^H]PGE_2 _remaining in the ipsilateral cerebrum (29.3 ± 5.6% and 25.2 ± 6.6%, respectively; Figure 4).

## Discussion

The present study demonstrated that intraperitoneal LPS administration resulted in attenuation of PGE_2 _elimination from the brain across the BBB (Figure 1A). The elimination rate in LPS-treated mice was decreased to 13.0% of that in saline-treated mice. Intraperitoneal administration of LPS at 3 mg/kg was reported to induce severe inflammatory reaction (sepsis syndrome) in mice [27,28]. Thus, it is suggested that PGE_2 _elimination across the BBB might be attenuated in the case of severe bacterial infection. These results indicate that the increase of cerebral concentration of endogenous PGE_2 _in LPS-induced severe inflammation reflects not only induction of PGE_2_-synthesizing enzymes, such as COX-2 and microsomal PGES, in the brain [18,29], but also potent inhibition of PGE_2 _elimination from the brain.

In general, it is considered that the integrity of the BBB is disrupted by LPS administration [13,30]. On the other hand, the transport of BBB-impermeable compounds, such as sodium fluorescein and serum albumin, was not significantly changed by LPS administration [31,32]. There was no significant variation in the percentage of [^14^C]inulin recovery between LPS-treated mice and saline-treated mice in our study. Thus, it is suggested that the attenuation of PGE_2 _elimination across the BBB by intraperitoneal 3.0 mg/kg LPS administration (Figure 1A) reflects the reduction of PGE_2 _efflux transport capacity at the BBB, but not disruption of the BBB.

We previously reported that Mrp4 was involved in PGE_2 _elimination from the brain across the BBB in normal mice [6]. It has been reported that LPS administration alters the protein expression and function of several transporters, such as P-glycoprotein, at the BBB [30]. However, in LPS-treated mice, the Mrp4 protein expression level was unchanged in brain capillaries, compared with that in saline-treated mice (Table 2). Although the protein expression of Oat3 and Oatp1a4 in brain capillaries was significantly decreased by 25.7% and 39.0%, respectively, in LPS-treated mice (Table 2), these changes of Oat3 and Oatp1a4 protein expression are insufficient to fully account for the 87.0% decrease of the PGE_2 _elimination rate. Furthermore, benzylpenicillin and digoxin treatment did not affect PGE_2 _elimination from the brain (compare Figure 3 and our previous results [6]). Under the conditions we used, Oat3 and Oatp1a4 would be inhibited by approximately 71% and 78%, respectively [33,34]. Therefore, these results suggest that Oat3 and Oatp1a4 do not play significant roles in the PGE_2 _elimination. Consequently, the attenuation of PGE_2 _elimination cannot be explained simply in terms of changes in protein expression of relevant transporters at the BBB.

In the present study, we determined protein expression levels in whole capillary endothelial cells, but not in plasma membrane fraction. Therefore, a possible explanation of our results is decreased protein levels of transporter(s) on the plasma membrane owing to induction of internalization. It has been reported that LPS treatment decreases the plasma membrane localization of Pgt at the apical membrane of rat primary-cultured cerebral endothelial cells [35]. On the other hand, the protein expression level of Pgt was below the detection limit in isolated brain capillaries of either saline- or LPS-treated mice (Table 2). It is suggested that the protein expression level of Pgt at the BBB is low compared with that of other organic anion transporters, and so the alteration of plasma membrane localization of Pgt is unlikely to have a major influence on PGE_2 _transport across the BBB. Another possibility is a change in the molecular transport activity as a result of protein modification. It has been reported that LPS treatment decreased P-glycoprotein activity, without changing its protein expression, via protein kinase C activation [36-38].

It is also conceivable that increased endogenous PGE_2 _inhibited the elimination of injected [^3^H]PGE_2 _from the brain. It was reported that endogenous PGE_2 _in the brain after 1 mg/kg LPS administration is increased to approximately 28 nM from 0.2 nM [14], and intraperitoneal LPS administration at 40 mg/kg increases the cerebral PGE_2 _concentration to approximately 141 nM [15]. Moreover, it has been reported that expression of COX-2 and microsomal PGES-1 in mouse brain capillaries is increased by LPS administration [16,31]. In rats, PGE_2_-derived immunoreactivity in brain capillaries of cerebral cortex is markedly increased by intravenous administration of 2.5 mg/kg LPS, compared with saline control [39]. Therefore, there is a possibility that increased endogenous PGE_2 _in brain capillary endothelial cells following LPS administration may inhibit PGE_2 _transport via Mrp4 at the luminal membrane of brain capillary endothelial cells.

[^3^H]PGE_2 _elimination across the BBB in LPS-treated mice was inhibited by co-administration of 3 mM unlabeled PGE_2 _(Figure 1B). The percentage of [^3^H]PGE_2 _remaining at 40 min was increased to 114% by 3 mM unlabeled PGE_2 _co-administration (Figure 1B), and this value was identical to the y-intercept of the time-profile of the percentage of [^3^H]PGE_2 _remaining in LPS-treated mice (108%, Figure 1A). Thus, it is suggested that carrier-mediated PGE_2 _efflux transport is partially retained in LPS-treated mice, and inhibition of PGE_2 _efflux transport by drug administration could lead to the disappearance of PGE_2 _elimination across the BBB. Intracerebral and intravenous pre-administration of cefmetazole also inhibited PGE_2 _elimination across the BBB in LPS-treated mice (Figure 2 and Table 3), suggesting that cefmetazole administration further inhibits PGE_2 _elimination from the brain in this mouse model of inflammation. Cefmetazole is known to induce fever [23], and fever is associated with increased PGE_2 _in the brain [8]. Therefore, the adverse effects of cefmetazole may be related to its inhibitory effect on PGE_2 _elimination, which is expected to result in increased cerebral accumulation of PGE_2_. The plasma unbound concentration of cefmetazole in mice can be calculated as 251 μM after intravenous administration of 200 mg/kg cefmetazole from the reported values of the volume of distribution of cefmetazole, unbound fraction of drug in plasma and half-life in circulating blood [40,41]. In humans, cefmetazole is intravenously administered at the dosage of 2 g/human, and the maximum serum unbound concentration of cefmetazole was reported as 80.0 μM at this dose [40,42]. In addition, the IC_50 _value of cefmetazole for Mrp4-mediated PGE_2 _transport was reported to be 10 μM [6]. Because the unbound concentration of cefmetazole in the circulating blood of both mouse and human is higher than this IC_50 _value, it is possible that the inhibition of PGE_2 _elimination across the BBB following intravenous administration of cefmetazole can occur in humans.

Cefmetazole in the circulation must enter brain capillary endothelial cells in order to inhibit MRP4-mediated PGE_2 _transport at the luminal membrane of the BBB. We found that the inhibitory effect of cefmetazole was blocked when Oatp1a4 was inhibited by intravenous administration of amiodarone (Figure 4), suggesting that Oatp1a4 mediates the entry of cefmetazole from the circulating blood into endothelial cells. This raises the possibility that co-administration of other Oatp1a4-substrate drugs might decrease the cerebral adverse effects of cefmetazole via interaction at Oatp1a4 on the luminal membrane of brain capillary endothelial cells.

Either intracerebral or intravenous cefazolin inhibited PGE_2 _elimination in mice not treated with LPS [6], and intracerebral administration of benzylpenicillin and digoxin suppressed the inhibitory effect of intracerebrally administered cefazolin (Figure 3). Intravenous administration of amiodarone also suppressed the inhibitory effect of intravenous administered cefazolin (Figure 4). These results suggest that cefazolin enters brain capillary endothelial cells from the brain via Oat3 and/or Oatp1a4, and from the circulation via Oatp1a4.

In LPS-treated mice, cefmetazole inhibited PGE_2 _elimination across the BBB, whereas cefazolin did not (Figure 2). After intravenous administration of 200 mg/kg cefazolin, the plasma unbound concentration of cefazolin in mice reaches 445 μM, as calculated from the volume of distribution of cefazolin, unbound fraction of drug in plasma and half-life in circulating blood [40,41]. This value is 5.5-fold higher than the Km value (80.9 μM) of Mrp4-mediated cefazolin transport [43]. On the other hand, cefazolin must be taken up into brain capillary endothelial cells from circulating blood in order to inhibit Mrp4-mediated PGE_2 _transport at the luminal membrane of the BBB. The affinity of cefmetazole for Mrp4 is 8.1-fold higher than that of cefazolin [6,43], suggesting that the local concentration of cefazolin in brain capillary endothelial cells may not be sufficiently high, compared with that of cefmetazole, to inhibit Mrp4. This seems reasonable, because expression of Oatp1a4 protein, which mediates cefazolin uptake into brain capillary endothelial cells from the circulating blood, is decreased in LPS-treated mice, so the local concentration of cefazolin in brain capillary endothelial cells may not be sufficiently high to inhibit Mrp4 at the luminal membrane of the BBB.

The inhibitory effect of drugs on PGE_2 _elimination from the brain was different in LPS-treated mice from that in untreated mice. Intracerebral 5 mM pre-administration of ceftriaxone, cefazolin, indomethacin and ketoprofen, which inhibit PGE_2 _elimination across the BBB in mice not treated with LPS [6], did not significantly inhibit PGE_2 _elimination in LPS-treated mice (Table 3). Furthermore, intravenous administration of cefazolin had little effect in LPS-treated mice (Figure 2), although intravenous administration of cefazolin showed a significant inhibitory effect on PGE_2 _elimination in mice not treated with LPS [6]. In contrast, intracerebral and intravenous administration of cefmetazole inhibited PGE_2 _elimination in either LPS-treated or -untreated mice. These results suggest that the PGE_2 _elimination process consists of a cefmetazole-sensitive process and a cefazolin-sensitive process, and LPS treatment mainly suppresses the cefazolin-sensitive process. Further study will be needed to clarify in detail the mechanism of PGE_2 _elimination from the brain and its regulation by LPS treatment, in order to understand the regulatory mechanisms of cerebral PGE_2 _levels and the mechanisms of the cerebral adverse effects of antibiotics.

## Conclusion

Our results show that PGE_2 _elimination from the brain across the mouse BBB is attenuated in LPS-induced inflammation, and intravenous administration of cefmetazole further increases the inhibition of PGE_2 _elimination. These findings indicate that the possibility of cerebral adverse effects should be carefully taken into account when using antibiotics to treat severe bacterial infections.

## List of abbreviations

BBB: blood-brain barrier; BEI: brain efflux index; CNS: central nervous system; COX: cyclooxygenase; DMSO: dimethylsulfoxide; ECF: extracellular fluid; HPLC: high-performance liquid chromatography; LC-MS/MS: liquid chromatography-tandem mass spectrometry; LPS: lipopolysaccharide; MRM: multiple reaction monitoring; MRP4: multidrug resistance-associated protein 4; NSAIDs: nonsteroidal anti-inflammatory drugs; Oat3: organic anion transporter 3; Oatp: organic anion transporting polypeptide; PGE_2_: prostaglandin E_2_; Pgt: prostaglandin transporter; PGES: prostaglandin E synthase; S2: secondary somatosensory cortex

## Competing interests

The authors declare that they have no competing interests.

## Authors' contributions

SA carried out the *in vivo *animal studies and manuscript preparation. YU carried out the quantification of transporter protein amounts in brain capillaries. SO and MT helped to draft the manuscript. TT and KH supervised the study design and manuscript preparation. All authors read and approved the final manuscript.

## References

[B1] Ohtsuki S Terasaki T Contribution of carrier-mediated transport systems to the blood-brain barrier as a supporting and protecting interface for the brain; importance for CNS drug discovery and development Pharm Res 2007 24 1745 1758 10.1007/s11095-007-9374-5 17619998

[B2] Leggas M Adachi M Scheffer GL Sun D Wielinga P Du G Mercer KE Zhuang Y Panetta JC Johnston B Mrp4 confers resistance to topotecan and protects the brain from chemotherapy Mol Cell Biol 2004 24 7612 7621 10.1128/MCB.24.17.7612-7621.2004 15314169 PMC506999

[B3] Mori S Takanaga H Ohtsuki S Deguchi T Kang YS Hosoya K Terasaki T Rat organic anion transporter 3 (rOAT3) is responsible for brain-to-blood efflux of homovanillic acid at the abluminal membrane of brain capillary endothelial cells J Cereb Blood Flow Metab 2003 23 432 440 12679720 10.1097/01.WCB.0000050062.57184.75

[B4] Ose A Kusuhara H Endo C Tohyama K Miyajima M Kitamura S Sugiyama Y Functional characterization of mouse organic anion transporting peptide 1a4 in the uptake and efflux of drugs across the blood-brain barrier Drug Metab Dispos 2010 38 168 176 10.1124/dmd.109.029454 19833843

[B5] Ohtsuki S Ito S Matsuda A Hori S Abe T Terasaki T Brain-to-blood elimination of 24S-hydroxycholesterol from rat brain is mediated by organic anion transporting polypeptide 2 (oatp2) at the blood-brain barrier J Neurochem 2007 103 1430 1438 10.1111/j.1471-4159.2007.04901.x 17868302

[B6] Akanuma S Hosoya K Ito S Tachikawa M Terasaki T Ohtsuki S Involvement of multidrug resistance-associated protein 4 in efflux transport of prostaglandin E_2 _across mouse blood-brain barrier and its inhibition by intravenous administration of cephalosporins J Pharmacol Exp Ther 2010 333 912 919 10.1124/jpet.109.165332 20194529

[B7] Alix E Schmitt C Strazielle N Ghersi-Egea JF Prostaglandin E_2 _metabolism in rat brain: Role of the blood-brain interfaces Cerebrospinal Fluid Res 2008 5 5 10.1186/1743-8454-5-5 18318891 PMC2292143

[B8] Sugimoto Y Narumiya S Prostaglandin E receptors J Biol Chem 2007 282 11613 11617 17329241 10.1074/jbc.R600038200

[B9] Chen C Bazan NG Endogenous PGE_2 _regulates membrane excitability and synaptic transmission in hippocampal CA1 pyramidal neurons J Neurophysiol 2005 93 929 941 15653788 10.1152/jn.00696.2004

[B10] Kurokawa M Imakita M Kumeda CA Shiraki K Cascade of fever production in mice infected with influenza virus J Med Virol 1996 50 152 158 10.1002/(SICI)1096-9071(199610)50:2<152::AID-JMV8>3.0.CO;2-9 8915881

[B11] Nguyen MD Julien JP Rivest S Innate immunity: the missing link in neuroprotection and neurodegeneration? Nat Rev Neurosci 2002 3 216 227 10.1038/nrn752 11994753

[B12] Erickson MA Banks WA Cytokine and chemokine responses in serum and brain after single and repeated injections of lipopolysaccharide: Multiplex quantification with path analysis Brain Behav Immun in press10.1016/j.bbi.2011.06.006 PMC3389494 21704698

[B13] Oshima S Nemoto E Kuramochi M Saitoh Y Kobayashi D Penetration of oseltamivir and its active metabolite into the brain after lipopolysaccharide-induced inflammation in mice J Pharm Pharmacol 2009 61 1397 1400 19814874 10.1211/jpp/61.10.0018

[B14] Golovko MY Murphy EJ An improved LC-MS/MS procedure for brain prostanoid analysis using brain fixation with head-focused microwave irradiation and liquid-liquid extraction J Lipid Res 2008 49 893 902 10.1194/jlr.D700030-JLR200 18187404

[B15] Sapirstein A Saito H Texel SJ Samad TA O'Leary E Bonventre JV Cytosolic phospholipase A_2_α regulates induction of brain cyclooxygenase-2 in a mouse model of inflammation Am J Physiol Regul Integr Comp Physiol 2005 288 R1774 1782 10.1152/ajpregu.00815.2004 15718387

[B16] Yamagata K Matsumura K Inoue W Shiraki T Suzuki K Yasuda S Sugiura H Cao C Watanabe Y Kobayashi S Coexpression of microsomal-type prostaglandin E synthase with cyclooxygenase-2 in brain endothelial cells of rats during endotoxin-induced fever J Neurosci 2001 21 2669 2677 11306620 10.1523/JNEUROSCI.21-08-02669.2001 PMC6762538

[B17] Bastos GN Moriya T Inui F Katura T Nakahata N Involvement of cyclooxygenase-2 in lipopolysaccharide-induced impairment of the newborn cell survival in the adult mouse dentate gyrus Neuroscience 2008 155 454 462 10.1016/j.neuroscience.2008.06.020 18616986

[B18] Inoue W Somay G Poole S Luheshi GN Immune-to-brain signaling and central prostaglandin E_2 _synthesis in fasted rats with altered lipopolysaccharide-induced fever Am J Physiol Regul Integr Comp Physiol 2008 295 R133 143 10.1152/ajpregu.90335.2008 18480240 PMC2494823

[B19] Kim SY Chang YJ Cho HM Hwang YW Moon YS Non-steroidal anti-inflammatory drugs for the common cold Cochrane Database Syst Rev 2009 CD006362 10.1002/14651858.CD006362.pub2 19588387

[B20] Hulscher ME Grol RP van der Meer JW Antibiotic prescribing in hospitals: a social and behavioural scientific approach Lancet Infect Dis 2010 10 167 175 10.1016/S1473-3099(10)70027-X 20185095

[B21] Nilwarangkoon S Anzai N Shiraya K Yu E Islam R Cha SH Onozato ML Miura D Jutabha P Tojo A Role of mouse organic anion transporter 3 (mOat3) as a basolateral prostaglandin E_2 _transport pathway J Pharmacol Sci 2007 103 48 55 10.1254/jphs.FP0060816 17220594

[B22] Taogoshi T Nomura A Murakami T Nagai J Takano M Transport of prostaglandin E_1 _across the blood-brain barrier in rats J Pharm Pharmacol 2005 57 61 66 15638994 10.1211/0022357055173

[B23] Jones RN Cefmetazole (CS-1170), a "new" cephamycin with a decade of clinical experience Diagn Microbiol Infect Dis 1989 12 367 379 10.1016/0732-8893(89)90106-5 2692950

[B24] Yamaoka K Tanigawara Y Nakagawa T Uno T A pharmacokinetic analysis program (MULTI) for microcomputer J Pharmacobiodyn 1981 4 879 885 7328489 10.1248/bpb1978.4.879

[B25] Uchida Y Ohtsuki S Katsukura Y Ikeda C Suzuki T Kamiie J Terasaki T Quantitative targeted absolute proteomics of human blood-brain barrier transporters and receptors J Neurochem 2011 117 333 345 10.1111/j.1471-4159.2011.07208.x 21291474

[B26] Kamiie J Ohtsuki S Iwase R Ohmine K Katsukura Y Yanai K Sekine Y Uchida Y Ito S Terasaki T Quantitative atlas of membrane transporter proteins: development and application of a highly sensitive simultaneous LC/MS/MS method combined with novel in-silico peptide selection criteria Pharm Res 2008 25 1469 1483 10.1007/s11095-008-9532-4 18219561

[B27] Warren HS Editorial: Mouse models to study sepsis syndrome in humans J Leukoc Biol 2009 86 199 201 10.1189/jlb.0309210 19643738

[B28] McCuskey RS McCuskey PA Urbaschek R Urbaschek B Species differences in Kupffer cells and endotoxin sensitivity Infection & Immunity 1984 45 278 280 6376358 10.1128/iai.45.1.278-280.1984 PMC263314

[B29] Inoue W Matsumura K Yamagata K Takemiya T Shiraki T Kobayashi S Brain-specific endothelial induction of prostaglandin E_2 _synthesis enzymes and its temporal relation to fever Neurosci Res 2002 44 51 61 10.1016/S0168-0102(02)00083-4 12204293

[B30] Salkeni MA Lynch JL Otamis-Price T Banks WA Lipopolysaccharide impairs blood-brain barrier P-glycoprotein function in mice through prostaglandin- and nitric oxide-independent pathways J Neuroimmune Pharmacol 2009 4 276 282 10.1007/s11481-008-9138-y 19039663 PMC2802264

[B31] Chung DW Yoo KY Hwang IK Kim DW Chung JY Lee CH Choi JH Choi SY Youn HY Lee IS Won MH Systemic administration of lipopolysaccharide induces cyclooxygenase-2 immunoreactivity in endothelium and increases microglia in the mouse hippocampus Cell Mol Neurobiol 2010 30 531 541 10.1007/s10571-009-9477-0 19908141 PMC11498804

[B32] Pan W Hsuchou H Yu C Kastin AJ Permeation of blood-borne IL15 across the blood-brain barrier and the effect of LPS J Neurochem 2008 106 313 319 10.1111/j.1471-4159.2008.05390.x 18384647 PMC3939609

[B33] van Montfoort JE Schmid TE Adler ID Meier PJ Hagenbuch B Functional characterization of the mouse organic-anion-transporting polypeptide 2 Biochim Biophys Acta 2002 1564 183 188 10.1016/S0005-2736(02)00445-5 12101011

[B34] Ohtsuki S Kikkawa T Mori S Hori S Takanaga H Otagiri M Terasaki T Mouse reduced in osteosclerosis transporter functions as an organic anion transporter 3 and is localized at abluminal membrane of blood-brain barrier J Pharmacol Exp Ther 2004 309 1273 1281 10.1124/jpet.103.063370 14762099

[B35] Kis B Isse T Snipes JA Chen L Yamashita H Ueta Y Busija DW Effects of LPS stimulation on the expression of prostaglandin carriers in the cells of the blood-brain and blood-cerebrospinal fluid barriers J Appl Physiol 2006 100 1392 1399 16322371 10.1152/japplphysiol.01259.2005

[B36] Fattori S Becherini F Cianfriglia M Parenti G Romanini A Castagna M Human brain tumors: multidrug-resistance P-glycoprotein expression in tumor cells and intratumoral capillary endothelial cells Virchows Archiv 2007 451 81 87 10.1007/s00428-007-0401-z 17593388

[B37] Hartz AM Bauer B Fricker G Miller DS Rapid modulation of P-glycoprotein-mediated transport at the blood-brain barrier by tumor necrosis factor-α and lipopolysaccharide Mol Pharmacol 2006 69 462 470 16278373 10.1124/mol.105.017954

[B38] Bauer B Hartz AM Miller DS Tumor necrosis factor α and endothelin-1 increase P-glycoprotein expression and transport activity at the blood-brain barrier Mol Pharmacol 2007 71 667 675 17132686 10.1124/mol.106.029512

[B39] Van Dam AM Brouns M Man AHW Berkenbosch F Immunocytochemical detection of prostaglandin E_2 _in microvasculature and in neurons of rat brain after administration of bacterial endotoxin Brain Res 1993 613 331 336 10.1016/0006-8993(93)90922-A 8186987

[B40] Komiya M Kikuchi Y Tachibana A Yano K Pharmacokinetics of new broad-spectrum cephamycin, YM09330, parenterally administered to various experimental animals Antimicrob Agents Chemother 1981 20 176 183 6945066 10.1128/aac.20.2.176 PMC181660

[B41] Lin JH Species similarities and differences in pharmacokinetics Drug Metab Dispos 1995 23 1008 1021 8654187

[B42] Ko H Cathcart KS Griffith DL Peters GR Adams WJ Pharmacokinetics of intravenously administered cefmetazole and cefoxitin and effects of probenecid on cefmetazole elimination Antimicrob Agents Chemother 1989 33 356 361 2729930 10.1128/aac.33.3.356 PMC171493

[B43] Ci L Kusuhara H Adachi M Schuetz JD Takeuchi K Sugiyama Y Involvement of MRP4 (ABCC4) in the luminal efflux of ceftizoxime and cefazolin in the kidney Mol Pharmacol 2007 71 1591 1597 10.1124/mol.106.031823 17344354

